# A Benchmark of Unsupervised Circadian Phase Inference Methods for Bulk Transcriptomic Data

**DOI:** 10.64898/2026.10.08.757747

**Published:** 2026-10-15

**Authors:** Souparna Purohit, Gregory R. Grant, Thomas G. Brooks

## Abstract

Circadian phase can be inferred from gene expression when sample collection times are unavailable, but the available methods have not been systematically compared. We compared ten unsupervised phase inference methods and two clock-gene baselines on 25 datasets with reference times (16 real datasets from five studies and 9 simulations). The primary measure was the aligned circular mean absolute error (MAE), with a permutation test for significance, circular correlations and gene-level reconstruction of rhythmicity, acrophase and amplitude were secondary outcomes. CHIRAL had the lowest mean MAE (1.80 h, 95% confidence interval 1.21-2.97 h from resampling study families). The two clock-gene baselines (2.15 and 2.20 h) were next, followed by TEMPO, CYCLOPS and CYCLOPS 2.0 (2.49-2.80 h), and the confidence intervals of these six overlapped substantially. The other six methods had mean errors of 3.91-4.86 h, compared with 5.08-5.23 h for permuted phases. Accuracy depended on the dataset and on the strength of clock-gene rhythms, and, for several methods, on the random seed and the adjustment for batch or covariates. When the inferred phases were used to analyse gene rhythms in 22 datasets, the four leading methods identified rhythmic genes with a median ROC AUC of 0.64-0.70 and recovered gene acrophases with a median error of 0.18-0.64 h. In two mouse datasets without collection times, agreement with external reference acrophases differed between methods and tissues.

